# Short- and Long-Term Prognosis of Intravascular Ultrasound-Versus Angiography-Guided Percutaneous Coronary Intervention: A Meta-Analysis Involving 24,783 Patients

**DOI:** 10.1155/2021/6082581

**Published:** 2021-10-15

**Authors:** Qun Zhang, Bailu Wang, Yu Han, Shukun Sun, Ruijuan Lv, Shujian Wei

**Affiliations:** ^1^Department of Emergency and Chest Pain Center, Qilu Hospital, Cheeloo College of Medicine, Shandong University, Jinan, Shandong 250012, China; ^2^Clinical Research Center for Emergency and Critical Care Medicine of Shandong Province, Institute of Emergency and Critical Care Medicine of Shandong University, Qilu Hospital, Cheeloo College of Medicine, Shandong University, Jinan, Shandong 250012, China; ^3^Key Laboratory of Emergency and Critical Care Medicine of Shandong Province, Key Laboratory of Cardiopulmonary-Cerebral Resuscitation Research of Shandong Province, Qilu Hospital, Cheeloo College of Medicine, Shandong University, Jinan, Shandong 250012, China; ^4^The Key Laboratory of Cardiovascular Remodeling and Function Research, Chinese Ministry of Education, Chinese Ministry of Health and Chinese Academy of Medical Sciences, The State and Shandong Province Joint Key Laboratory of Translational Cardiovascular Medicine, Qilu Hospital, Cheeloo College of Medicine, Shandong University, Jinan, Shandong 250012, China; ^5^Clinical Trial Center, Qilu Hospital, Cheeloo College of Medicine, Shandong University, Jinan, Shandong,250012, China

## Abstract

**Background:**

Intravascular ultrasound (IVUS) guided percutaneous coronary intervention (PCI) has potential benefits. This meta-analysis aimed to explore whether IVUS-guided PCI had better short- and long-term prognoses than angiography-guided PCI.

**Methods:**

We retrieved studies from PubMed, Embase, and Cochrane Library. Clinical trials including retrospective and randomized controlled trials (RCTs) that compared IVUS-guided PCI with angiography-guided PCI were included. The patients were followed up after operation at 30 days, 1 year, 2 years, and 3 years. The clinical outcomes were target lesion revascularization (TLR), target vessel revascularization (TVR), and MACEs, including stent thrombosis (ST), myocardial infarction (MI), cardiac death, and all-cause death. The study population included patients with MI, coronary bifurcation lesions, short or long lesions, and unprotected left main coronary artery stenosis (ULMCA). The quality of retrospective trials was evaluated using the Newcastle–Ottawa Scale, and the quality of randomized controlled trials was evaluated using the Jadad score. A total of 20 clinical trials met the criteria. Three trials were randomized controlled trials, while 17 were retrospective trials.

**Results:**

A total of 24,783 patients were included. In observational trials, the OR of MACEs was 0.49 (95% CI: 0.38–0.62) in 30 days, 0.65 (95% CI: 0.58–0.73) in one year, 0.51 (95% CI: 0.36–0.71) in two years, and 0.45 (95% CI: 0.31–0.65) in three years. In patients with long coronary lesions, the OR of MACEs in 1 year was 0.64 (95% CI: 0.28–1.50). In patients with left main artery disease, the OR of MACEs in 3 years was 0.42 (95% CI: 0.26–0.67). Compared with angiography-guided PCI, IVUS-guided PCI was associated with a lower incidence of MACEs during the same following period.

**Conclusion:**

Compared with angiography-guided PCI, IVUS-guided PCI has better performance in reducing the occurrence of MACEs.

## 1. Introduction

Coronary artery disease (CAD) due to blockage or stenosis of the coronary arteries is a major cause of morbidity and mortality worldwide [1]. Coronary revascularization, including percutaneous coronary intervention (PCI) and coronary artery bypass graft (CABG), is the most effective treatment for CAD. PCI has been frequently performed because of its convenience and reduced risk of trauma [2]. However, PCI-related complications, including in-stent restenosis and stent thrombosis, limit its advantages. Thus, improving the procedural technologies of PCI is critical to the clinical outcomes of patients with CAD [3]. The clinical application of IVUS provides more accurate details of coronary lesions by comprehensively evaluating the structure of the coronary arteries [4, 5]. Since IVUS shows the whole coronary vessel wall and lumen, it facilitates the understanding of the pathophysiological process involved in coronary atherosclerosis [6].

The clinical benefits of IVUS-guided PCI were verified by several randomized controlled trials (RCTs). However, in clinical practice, the use of IVUS technology to guide PCI remains low, which may be related to the lack of clinical evidence. This study aimed to provide more detailed clinical evidence for IVUS to optimize PCI. In this meta-analysis, the clinical outcomes of major cardiovascular adverse events (MACEs) were compared between the IVUS-guided PCI group and the angiography-guided PCI group. We investigated the short- and long-term prognoses of IVUS-guided PCI in different populations by merging their follow-up time.

## 2. Materials and Methods

### 2.1. Retrieval Strategy

The relevant literature was retrieved by searching Embase, PubMed, Cochrane Controlled Trial Registry, and other online electronic databases. The search terms were as follows: “intravascular ultrasound,” “angiography,” and “percutaneous coronary intervention.” The purpose of our study was to compare the short- and long-term prognoses of the IVUS and angiography-guided PCI in CAD patients. Retrieved trials were further screened to identify studies that meet the criteria.

### 2.2. Inclusion and Exclusion Criteria

The inclusion criteria were as follows: (1) patients treated with IVUS-guided PCI as the experimental group; (2) patients treated with angiography-guided PCI as the control group; (3) randomized controlled trials or retrospective studies; and (4) no limit for the relevant population, including complex coronary artery disease, and left main artery disease.

### 2.3. Data Extraction, Quality Assessment, and Study Outcomes

Two researchers extracted data from articles that met the criteria. They subsequently summarized the basic characteristics of these articles, which included the name of the investigator, the date the article was published, study population, follow-up time, study design, and quality assessment score. The following data were then extracted: investigator's name, time of publication, and clinical outcomes. The primary outcomes were MACEs, including stent thrombosis, cardiac death, myocardial infarction, and all-cause death. The secondary outcomes were TLR and TVR. We analyzed the occurrence of clinical events in different follow-up times. Quality assessment was performed for studies that met the criteria. Two tools, the Jadad score and the Newcastle–Ottawa Scale (NOS), were used for quality assessment.

### 2.4. Statistical Analyses

All of the data were binary variables. Statistical analyses were performed using odds ratio (OR), risk ratio (RR), and 95% confidence interval (CI) to assess the risk of different surgical approaches. Heterogeneity was evaluated using the *Q* test and the *I*^2^ test. *P* < 0.1 or *I*^2^ > 50% corresponded to a greater heterogeneity. Data were analyzed using a random-effects model. The stability of the included studies was assessed by sensitivity analysis. Sensitivity analysis was performed by deleting one study and then repeating the meta-analysis. If *I*^2^ > 50%, we performed sensitivity and subgroup analyses. The full text of the trials that caused the heterogeneity of the analysis results and the explanation on whether to delete the article in the discussion section may be read. The Egger test and the funnel plots were used to assess potential bias. All operations were performed using Review Manager 5.3 software.

## 3. Results

### 3.1. Included Studies

**Figure 1:**
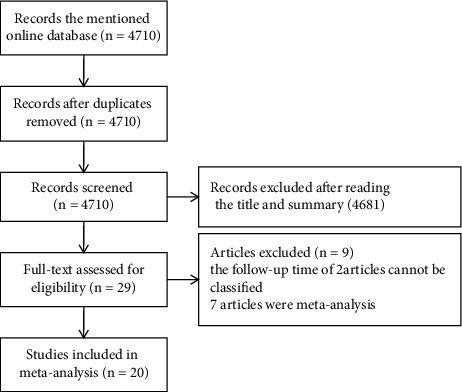
The flowchart of literature retrieval.

We searched related electronic databases, in which a total of 4,072 articles were retrieved. The full text, title, and abstract of the articles were read. Duplicate documents were deleted. In total, 29 articles were retained. The specific details of the 29 articles were discussed by all of the researchers. Among them, nine articles did not meet the inclusion criteria, seven articles were meta-analyses, and the follow-up time of the two other articles could not be classified. The flowchart of literature retrieval is shown in Figure 1.

A total of 20 clinical trials met the criteria [7–26]. Of the 20 trials, three were randomized controlled trials, while the other 17 were retrospective trials. Patients who were treated with IVUS-guided PCI belonged to the experimental group, while those who were treated with angiography-guided PCI belonged to the control group. The types of stents included drug-eluting stents and nondrug-eluting stents. The follow-up time of the six studies was 30 days; the follow-up time of 13 studies was one year; the follow-up time of five studies was 2 years; and the follow-up time of six studies was 3 years. The clinical endpoints in this trial were TLR, TVR, and MACEs, including ST, MI, cardiac death, and all-cause death. The populations of two studies involved patients with colonial bifurcation lesions; the population of one study was patients with complex lesions; the populations of two studies were patients with long coronary lesions; and the populations of six studies were patients with left main lesions. The basic characteristics of the articles that are included in the meta-analysis are summarized in Table 1.

### 3.2. Primary Outcomes

**Figure 2:**
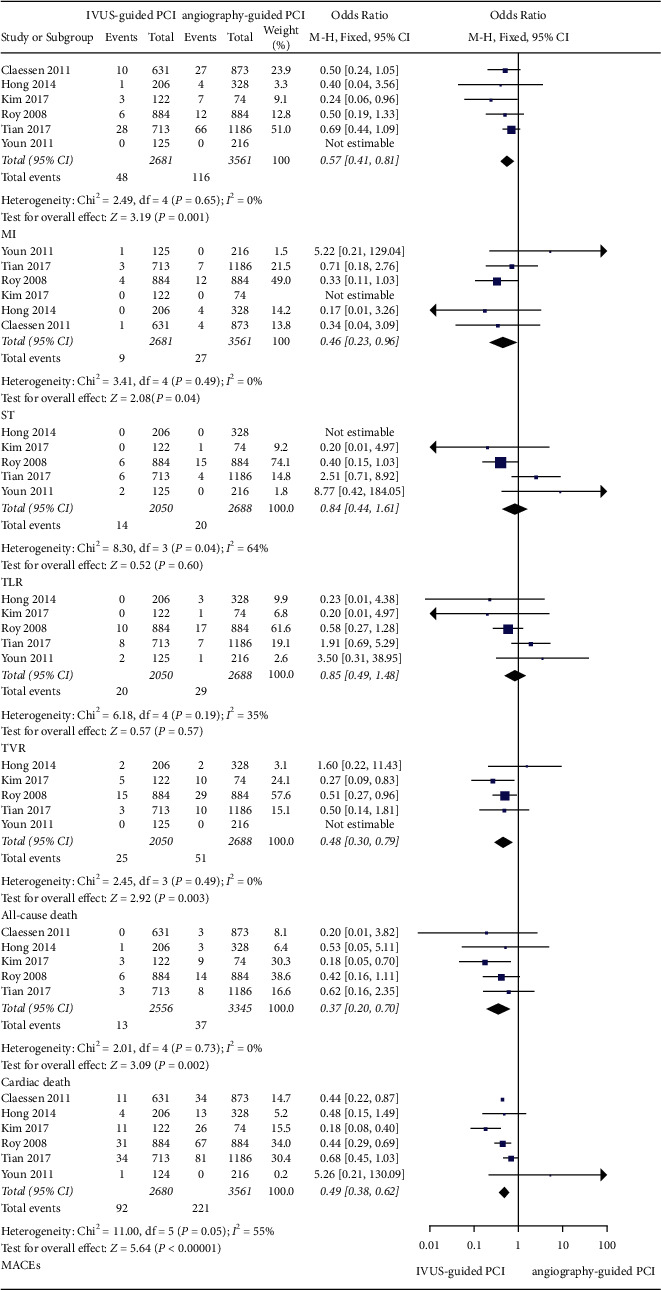
The forest plots of MACEs, TLR, and TVR in 30 days. MI: myocardial infarction; TLR: target lesion revascularization; TVR: target vessel revascularization; ST: stent thrombosis; MACEs: major adverse cardiovascular events.

**Figure 3:**
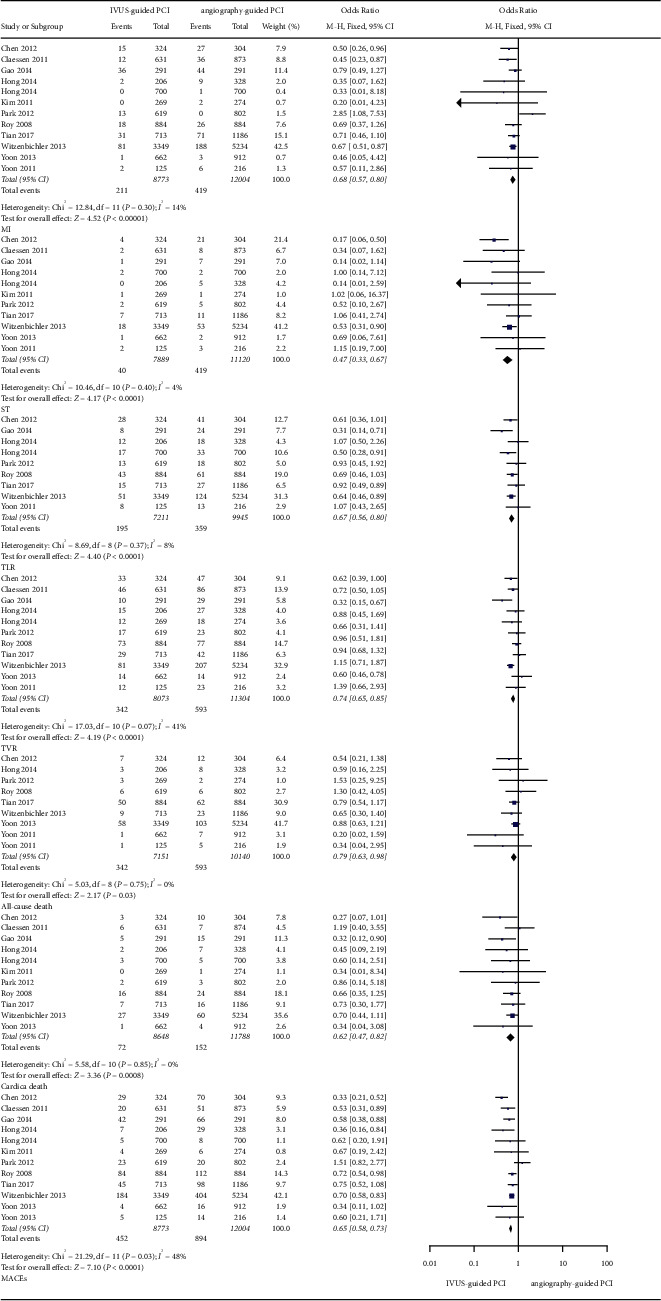
The forest plots of MACEs, TLR, and TVR in 1 year. MACEs: major adverse cardiovascular events.

**Figure 4:**
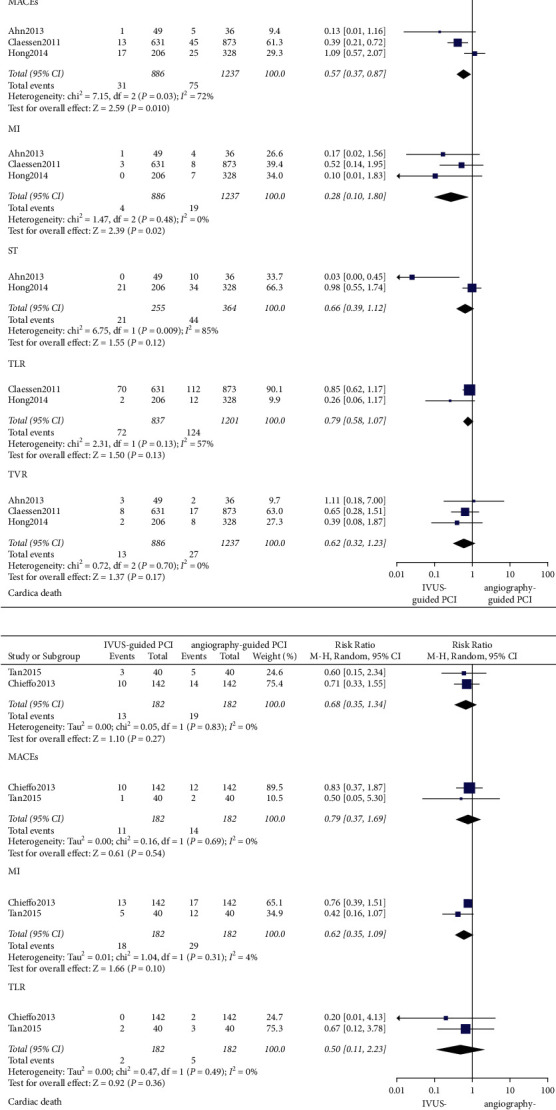
The forest plots of MACEs, TLR, and TVR in 2 years. ST: stent thrombosis; MI: myocardial infarction; TLR: target lesion revascularization; TVR: target vessel revascularization; MACEs: major adverse cardiovascular events.

**Figure 5:**
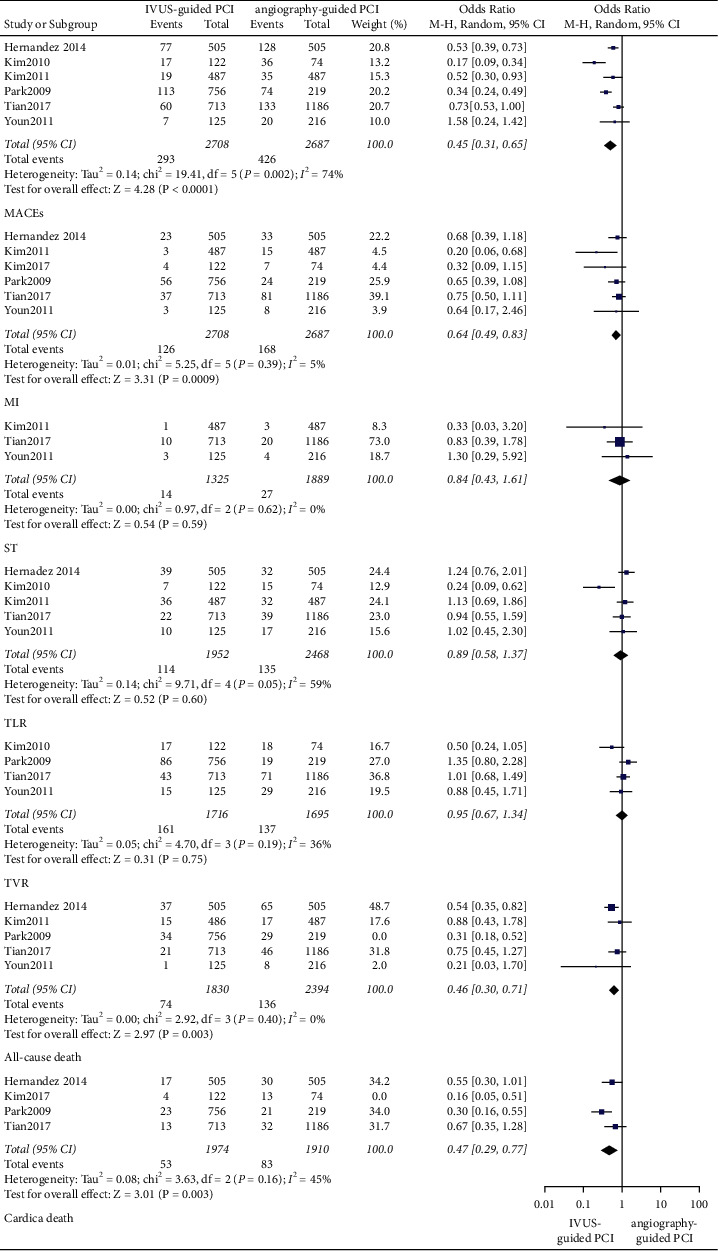
The forest plots of MACEs, TLR, and TVR in 3 years. MACEs: major adverse cardiovascular events; TVR: target vessel revascularization; TLR: target lesion revascularization.

In observational trials, after a 30-day follow-up period, it was found that IVUS-guided PCI was associated with a lower incidence of ST (OR: 0.46, 95% CI: 0.23–0.96, *P*=0.04, *I*^2^ = 0%), MI (OR: 0.57, 95% CI: 0.41–0.81, *P*=0.001, *I*^2^ = 0%), cardiac death (OR: 0.37, 95% CI: 0.20–0.70, *P*=0.002, *I*^2^ = 0%), all-cause death (OR: 0.48, 95% CI: 0.30–0.79; *P*=0.003, *I*^2^ = 0%), and MACEs (OR: 0.49, 95% CI: 0.38–0.62; *P* < 0.001, *I*^2^ = 55%) (Figure 2). After a 1-year follow-up period, the IVUS-guided PCI was associated with a lower incidence of ST (OR: 0.47, 95% CI: 0.33–0.67, *P* < 0.001, *I*^2^ = 4%), MI (OR: 0.68, 95% CI: 0.57–0.80, *P* < 0.001, *I*^2^ = 14%), cardiac death (OR: 0.62, 95% CI: 0.47–0.82, *P* < 0.001, *I*^2^ = 0%), all-cause death (OR: 0.79, 95% CI: 0.63–0.98, *P*=0.03, *I*^2^ = 0%), and MACEs (OR: 0.65, 95% CI: 0.58–0.73, *P* < 0.001, *I*^2^ = 48%) (Figure 3). At the 2-year follow-up, the IVUS-guided PCI was associated with a lower incidence of ST (OR: 0.28, 95% CI: 0.10–0.80, *P*=0.02, *I*^2^ = 0%), MI (OR: 0.57, 95% CI: 0.37–0.87, *P*=0.010, *I*^2^ = 72%), and MACEs (OR: 0.51, 95% CI: 0.36–0.71; *P* < 0.001, *I*^2^ = 0%) (Figure 4). At the 3-year follow-up, the IVUS-guided PCI was associated with a lower incidence of MI (OR: 0.64, 95% CI: 0.49–0.83, *P*=0.0009, *I*^2^ = 5%), cardiac death (OR: 0.41, 95% CI: 0.24–0.69, *P*=0.0009, *I*^2^ = 55%), all-cause death (OR: 0.54, 95% CI: 0.36–0.81, *P*=0.003, *I*^2^ = 53%), and MACEs (OR: 0.45, 95% CI: 0.31–0.65, *P* < 0.001, *I*^2^ = 74%) (Figure 5). In the randomized controlled trials, after a 2-year follow-up period, the incidence of MACEs (RR: 0.68, 95% CI: 0.35–1.34, *P*=0.27, *I*^2^ = 0%) was not significantly different between the IVUS-guided PCI and angiography-guided PCI (Figure 4).

**Figure 6:**
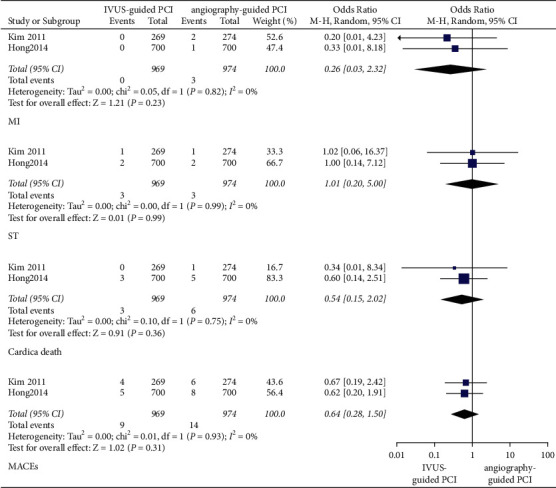
In patients with long coronary lesions, the forest plots of MACEs in 1 year. MACEs: major adverse cardiovascular events; MI: myocardial infarction; ST: stent thrombosis.

In patients with long coronary lesions, after a 1-year follow-up period, the incidence of MACEs (OR: 0.64, 95% CI: 0.28–1.50, *P*=0.31, *I*^2^ = 0%), cardiac death (OR: 0.54, 95% CI: 0.15–2.02, *P*=0.36, *I*^2^ = 0%), MI (OR: 0.26, 95% CI: 0.03–2.32, *P*=0.23, *I*^2^ = 0%), and ST (OR: 1.01, 95% CI: 0.20–5.00, *P*=0.99, *I*^2^ = 0%) was not significantly different between the IVUS-guided PCI and angiography-guided PCI (Figure 6).

**Figure 7:**
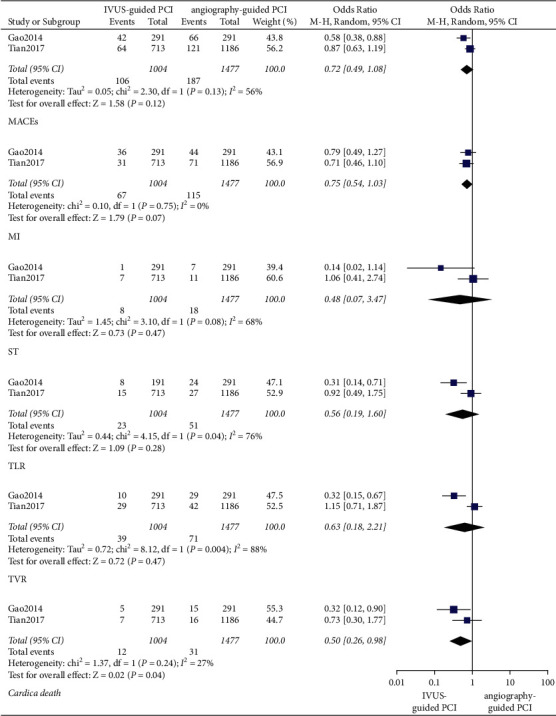
In patients with left main artery disease, the forest plots of MACEs in 1 year. TLR: target lesion revascularization; MI: myocardial infarction; TVR: target vessel revascularization; ST: stent thrombosis; MACEs: major adverse cardiovascular events.

**Figure 8:**
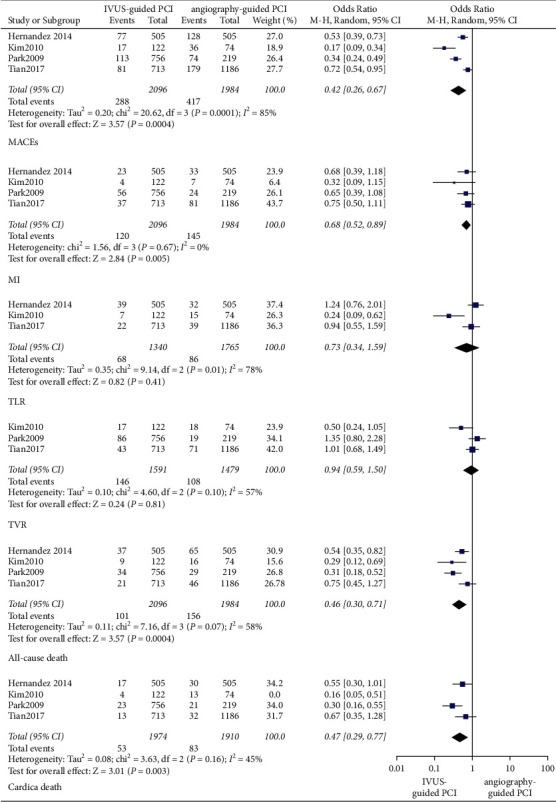
In patients with left main artery disease, the forest plots of MACEs, TLR, and TVR in 3 years. TLR: target lesion revascularization; TVR: target vessel revascularization; MACEs: major adverse cardiovascular events.

In patients with left main artery disease, at the 1-year follow-up, the IVUS-guided PCI was associated with a lower incidence of cardiac death (OR: 0.50, 95% CI: 0.26–0.98, *P*=0.04, *I*^2^ = 27%). As for MI (OR: 0.75, 95% CI: 0.54–1.03, *P*=0.08, *I*^2^ = 0%), ST (OR: 0.48, 95% CI: 0.07–3.47, *P*=0.47, *I*^2^ = 68%), and MACEs (OR: 0.72, 95% CI: 0.49–1.08, *P*=0.12, *I*^2^ = 56%), there was no significant difference between the IVUS-guided PCI and angiography-guided PCI (Figure 7). At the 3-year follow-up, the IVUS-guided PCI was associated with a lower incidence of all-cause death (OR: 0.46, 95% CI: 0.30–0.71, *P*=0.0004, *I*^2^ = 58%), cardiac death (OR: 0.41, 95% CI: 0.24–0.69, *P*=0.0009, *I*^2^ = 55%), MI (OR: 0.68, 95% CI: 0.52–0.88, *P*=0.004, *I*^2^ = 0%), and MACEs (OR: 0.42, 95% CI: 0.26–0.67, *P*=0.0004, *I*^2^ = 85%) (Figure 8).

### 3.3. Secondary Outcomes

In the observational trials, after a 30-day follow-up period, the incidence of TLR (OR: 0.84, 95% CI: 0.44–1.61, *P*=0.60, *I*^2^ = 64%) and TVR (OR: 0.85, 95% CI: 0.52–1.38, *P*=0.50, *I*^2^ = 19%) was not different between the IVUS-guided PCI and angiography-guided PCI (Figure 2). At the 1-year follow-up, the IVUS-guided PCI was associated with a lower incidence of TLR (OR: 0.67, 95% CI: 0.56–0.80, *P* < 0.001, *I*^2^ = 8%) and TVR (OR: 0.74, 95% CI: 0.65–0.85, *P* < 0.001, *I*^2^ = 41%) (Figure 3). At the 2-year follow-up, the incidence of TLR (OR: 0.66, 95% CI: 0.39–1.12, *P*=0.12, *I*^2^ = 85%) and TVR (OR: 0.79, 95% CI: 0.58–1.07, *P*=0.13, *I*^2^ = 57%) was not different between the IVUS-guided PCI and angiography-guided PCI (Figure 4). At the 3-year follow-up, the incidence of TLR (OR: 0.89, 95% CI: 0.58–1.37, *P*=0.60, *I*^2^ = 59%) and TVR (OR: 0.95, 95% CI: 0.67–1.34, *P*=0.75, *I*^2^ = 36%) was not significantly different between the IVUS-guided PCI and angiography-guided PCI (Figure 5). In the randomized controlled trials, at the 2-year follow-up, the incidence of TLR (RR: 0.62, 95% CI: 0.35–1.09, *P*=0.10, *I*^2^ = 4%) was not significantly different between the IVUS-guided PCI and angiography-guided PCI (Figure 4).

In patients with left main artery disease, at the 1-year follow-up, there were no significant differences between IVUS-guided PCI and angiography-guided PCI in terms of TLR (OR: 0.56, 95% CI: 0.19–1.60, *P*=0.28, *I*^2^ = 76%) and TVR (OR: 0.63, 95% CI: 0.18–2.21, *P*=0.47, *I*^2^ = 88%) (Figure 7). At the 3-year follow-up, the occurrence of TLR (OR: 0.73, 95% CI: 0.34–1.56, *P*=0.41, *I*^2^ = 78%) and TVR (OR: 0.94, 95% CI: 0.59–1.50, *P*=0.81, *I*^2^ = 57%) was not significantly different between the IVUS-guided PCI and angiography-guided PCI (Figure 8).

### 3.4. Bias Analysis

**Figure 9:**
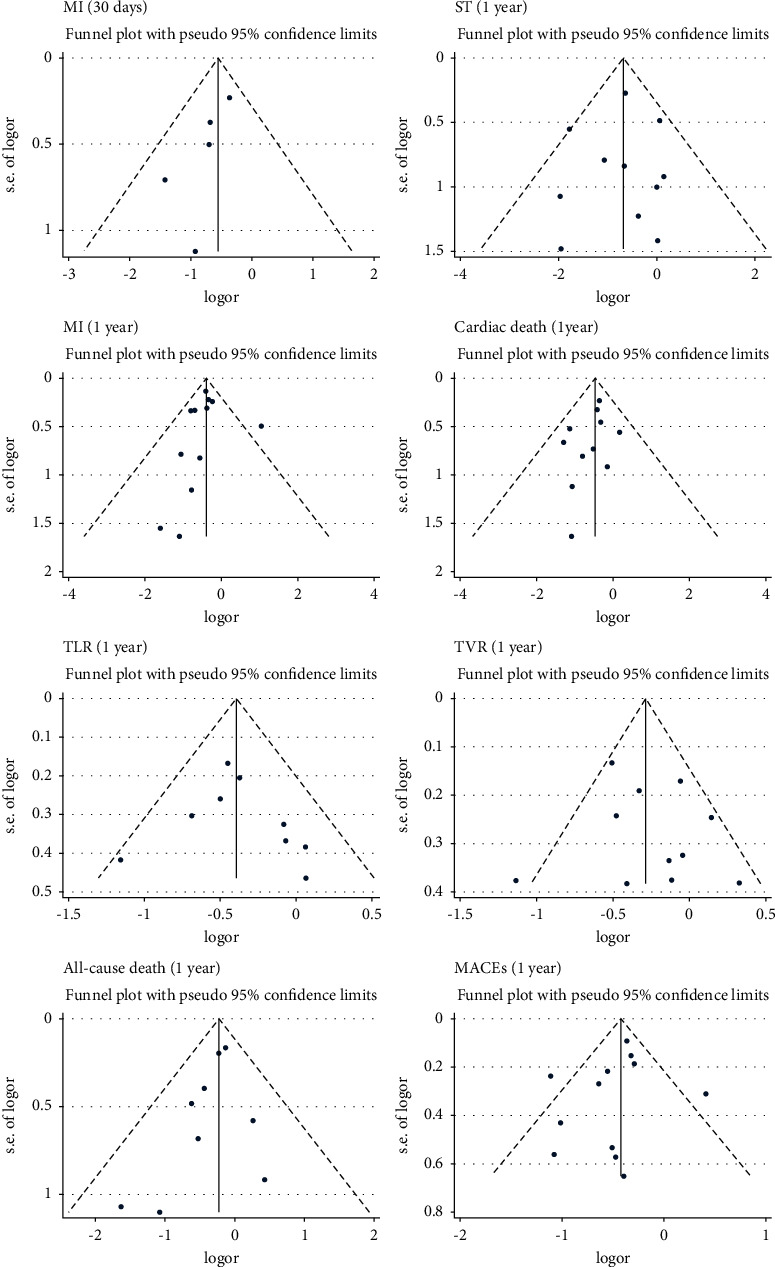
The funnel plots of MI (30 days), ST (1 year), MI (1 year), cardiac death (1 year), TLR (1 year), TVR (1 year), all-cause death (1 year), and MACEs (1 year). TLR: target lesion revascularization; TVR: target vessel revascularization; MACEs: major adverse cardiovascular events.

**Figure 10:**
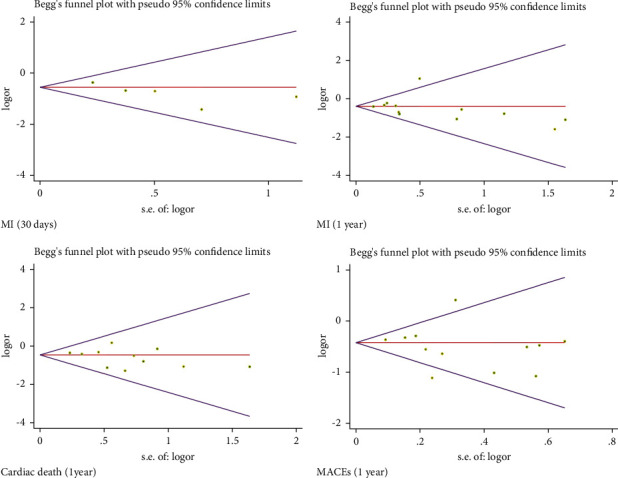
Begg's funnel plot of MI (30 days), MI (1 year), cardiac death (1 year), and MACEs (1 year). MI: myocardial infarction; MACEs: major adverse cardiovascular events.

We analyzed the bias of the related results. The funnel plots are shown in Figure 9. For the asymmetric funnel plots, Begg's and Egger's tests were performed. The results showed that there was no significant publication bias (Figure 10).

## 4. Discussion

A total of 20 clinical trials were included, of which three were randomized controlled and 17 were retrospective. In our analysis, compared with angiography-guided therapy, IVUS-guided therapy had a better long-term prognosis. In the short-term prognosis, IVUS-guided therapy also showed beneficial effects.

IVUS had been used for about 20 years in clinical practice. However, it has not been widely used due to the individual mode of practice, time pressure, and expenses [27]. IVUS can be used to evaluate plaque morphology, coronary artery dissection, and intramural hematoma. In addition, it has certain advantages in evaluating the anatomic severity of coronary artery disease. In the process of stent implantation, the use of IVUS reduces the occurrence of stent underexpansion. Besides, IVUS plays an important role in the evaluation of stent malapposition, tissue protrusion after stent placement, and coronary spasm [28, 29]. Although coronary angiography has always been the gold standard for coronary artery evaluation, it also has some limitations [30, 31]. For example, in patients with left main artery disease, overlap of the vessels may mask the left main artery lesion, which limits the role of angiography in assessing the severity of the lesion. However, for IVUS, significant stenosis can be accurately evaluated [32, 33]. The study conducted by Ye et al. reported that the positive predictive value of angiography was only 35.1% [5]. However, the application of IVUS did not reduce the incidence of TVR or TLR. This might be related to the low incidence of events and individual differences of interventional physicians [34]. This may explain why high heterogeneity happens to the result of TVR and TLR in this meta-analysis.

In this study, to eliminate the bias caused by the study design of the included studies, we analyzed the relevant MACEs of observational trials and randomized controlled trials, respectively. For RCTs, the forest plot results of MACEs showed no significant difference (Figure 4). In the IVUS guidance in RCTs, attention should be given to the occurrence of cardiac death and all-cause death in 30 days, 1 year, and 3 years, which is lower compared with that in angiography-guided PCI. Although IVUS-guided PCI has better performance in reducing the occurrence of mortality, we still cannot ignore the cost of IVUS-guided PCI. After spending a lot of treatment fees, who would benefit the most from IVUS guidance? This is an important issue that cannot be ignored, especially in developing countries. Therefore, it is necessary to identify those who would suffer. In this study, we performed a meta-analysis on the related MACEs in the population with long lesion disease, but the results showed no significant statistical difference. Moreover, IVUS showed beneficial effects for patients with left main disease, but only 2 studies were included in this meta-analysis. This suggests that we need to include more populations for meta-analysis in the future to determine patients who would benefit most from IVUS guidance despite the cost of treatment.

At the 3-year follow-up period, the result of MACE analysis showed a great heterogeneity. We then carried out a sensitivity analysis to evaluate the stability of the relevant research. After reading the full text of the article carefully and discussing it with all researchers, the reason for the large heterogeneity was related to the study design. There was great heterogeneity in the result of cardiac death. After the sensitivity analysis, the OR of cardiac death in 3 years was 0.47 (95% CI: 0.29–0.77, *P*=0.16, *I*^2^ = 45%). After carefully reading the full text and discussing with all of the researchers, the reason for the greater heterogeneity was related to the population differences [35]. The results of all-cause death also had a greater heterogeneity. We used the same method after deleting studies that caused greater heterogeneity. The OR of all-cause death at the 3-year follow-up was 0.64 (95% CI: 0.47–0.86, *P*=0.40, *I*^2^ = 0%). After reading the full text, we believed that the reasons for the greater heterogeneity were related to the study design and the heterogeneity of populations.

In previous meta-analyses, IVUS-guided treatment could reduce the incidence of MACEs in patients with complex lesions [36]. In this meta-analysis, IVUS-guided therapy played a better role in reducing the incidence of MACEs, TLR, and TVR [37]. The beneficial effects of IVUS were not limited to reducing the incidence of MACEs. It can also reduce clinical events such as stent thrombosis and death [38]. Moreover, IVUS-guided treatment played a beneficial role in reducing the incidence of acute myocardial infarction in patients with left main coronary artery disease [34]. However, we found that no one analyzed the long-term prognosis of IVUS-guided therapy. In this meta-analysis, we first classified the follow-up time of these studies and discussed the 30-day prognosis, 1-year prognosis, 2-year prognosis, and 3-year prognosis of IVUS-guided therapy. The results showed that IVUS-guided therapy had a better long-term prognosis. Interestingly, IVUS-guided therapy could reduce the incidence of TLR and TVR in 1 year, but there was no significant difference between the two strategies in 30 days, 2 years, and 3 years.

This meta-analysis proved that IVUS-guided PCI improved the short- and long-term prognoses of patients with PCI. Thus, we conclude that IVUS-guided therapy is superior to angiography-guided therapy in terms of reducing MACEs. However, we cannot deny the limitations of this meta-analysis. Only three of the studies were RCTs, while 17 were retrospective trials. Moreover, the number of studies related to the patients with long coronary artery disease and left main coronary artery disease was relatively small, leading to some degree of deviation. Thus, more clinical trials are needed to prove the accuracy of our results.

## 5. Conclusions

Compared with angiography-guided PCI, IVUS-guided PCI improves the short- and long-term prognoses of patients with PCI. In patients with long coronary lesions or left main artery disease, IVUS-guided PCI also manifests potential benefits.

## Figures and Tables

**Table 1:** The characteristics of included studies.

Study	Year	No. of participants	Study design	Population	Follow-up time	Quality assessment

Hong et al. [7]	2014	206/328	Observational	(1)	3 months, 1 year, 2 years	8
Kim et al. [8]	2011	487/487	Observational	(2)	3 years	8
Chieffo et al. [9]	2013	142/142	RCT	(3)	30 days, 2 years	7
Yoon et al. [10]	2013	662/912	Observational	(4)	1 year	8
Park et al. [11]	2012	619/802	Observational	(5)	1 year	8
Claessen et al. [12]	2011	631/873	Observational	(1)	30 days, 1 year, 2 years	9
Kim et al. [14]	2011	269/274	Observational	(6)	1 year	9
de la Torre Hernandez et al. [13]	2014	505/505	Observational	(7)	3 years	8
Ahn et al. [15]	2013	49/36	Observational	(8)	2 years	7
Chen et al. [16]	2012	324/304	Observational	(9)	1 year	8
Park et al. [21]	2009	756/219	Observational	(10)	3 years	9
Kim et al. [39]	2015	201/201	RCT	(11)	1 year	7
Witzenbichler et al. [40]	2013	3349/5234	Observational	(1)	1 year	9
Youn et al. [26]	2011	125/216	Observational	(12)	30 days, 1 year, 3 years	8
Roy et al. [22]	2008	884/884	Observational	(1)	30 days, 1 year	9
Gao et al. [17]	2014	291/291	Observational	(7)	1 year	9
Hong et al. [18]	2014	700/700	Observational	(6)	1 year	9
Kim et al. [20]	2017	122/74	Observational	(10)	30 days, 3 years	9
Tan et al. [23]	2015	40/40	RCT	(10)	2 years	6
Tian et al. [24]	2017	713/1186	Observational	(10)	30 days, 1 year, 3 years	9

RCT: randomized controlled trial; (1) patients were treated by DES; (2) patients with bifurcation lesions; (3) patients with coronary complex lesions; (4) patients with coronary short-length lesions; (5) patients were treated by PCI; (6) patients with coronary long lesions; (7) patients with coronary left main lesions; (8) patients with diffuse coronary artery disease; (9) patients with coronary bifurcation lesions; (10) patients with unprotected left main coronary artery lesions; (11) patients with chronic total occlusion; (12) patients with myocardial infarction.

## Data Availability

The data that support the findings of this study are available from the corresponding author upon reasonable request.
